# The effects of training with high‐speed interval running on muscle performance are modulated by slope

**DOI:** 10.14814/phy2.14656

**Published:** 2021-01-05

**Authors:** George Theofilidis, Gregory C. Bogdanis, Antonios Stavropoulos‐Kalinoglou, Argyro A. Krase, Themistoklis Tsatalas, Gary Shum, Giorgos K. Sakkas, Yiannis Koutedakis, Christina Karatzaferi

**Affiliations:** ^1^ Experimental Physiology & Therapeutic Exercise Laboratory Muscle Physiology and Mechanics Group CREHP School of Physical Education Sports Science and Nutrition University of Thessaly Trikala Greece; ^2^ School of Physical Education and Sports Science National and Kapodistrian University of Athens Dafni Greece; ^3^ Carnegie School of Sport Leeds Beckett University Leeds UK; ^4^ Experimental Physiology & Therapeutic Exercise Laboratory LIVE group CREHP School of Physical Education Sports Science and Nutrition University of Thessaly Trikala Greece; ^5^ Biomechanical Solutions Karditsa Greece; ^6^ Plymouth Marjon University Plymouth UK; ^7^ Cardiff Metropolitan University Cardiff UK; ^8^ Faculty of Arts University of Wolverhampton Wolverhampton UK

**Keywords:** concurrent training, fatigue, interval training, muscle power, optimization of training

## Abstract

We examined changes in selected muscle performance parameters after 8 weeks of interval training using two opposite running inclinations. We hypothesized that the uphill training will affect endurance muscle performance outcomes, whereas the downhill training will affect power muscle performance outcomes. Fourteen physically active volunteers were randomly assigned into either the Uphill group (UG; *n* = 7; uphill interval running at +10% incline) or the Downhill group (DG; *n* = 7; downhill interval running at −10% incline) and completed 16 training sessions. Each session consisted of ten 30 s treadmill runs at 90% of maximum aerobic speed (MAS) with a work to rest ratio of 1:2. Vertical jump performance, isometric (MVC) and isokinetic torque of knee extensors and flexors, and fatigue of knee extensors were evaluated pre and post‐training. Moreover, body composition (via bioimpedance) and vastus lateralis muscle architecture (via ultrasonography) were assessed pre and post‐training. Relative lean tissue mass, relative fat mass, and squat jump (cm) significantly (*p* < .05) changed from baseline values by +4.5 ± 4.0%, −11.5 ± 9.6%, and +9.5 ± 11.7%, respectively, only in the DG. Similarly, DG improved absolute values of knee extension rate of torque development and impulse (*p* < .05), whereas knee flexion peak torque angle significantly decreased in both groups (*p* < .05). On the other hand, the UG increased the number of repetitions achieved during the fatigue protocol and total work by 21.2 ± 32.6% and 13.8 ± 21.2%, respectively (*p* < .05). No differences were found between groups in muscle architecture. Introducing variations in slope during HIIT could be used to induce specific improvements toward muscle endurance or power performance characteristics.

## Graphical abstract

Trying for optimization of power performance and fatigue tolerance, the overall aim was to examine if by modulating the inclination of the running surface we could improve or maintain muscle strength/power characteristics. In conclusion, introducing variations in slope during HIIT could be used to induce specific improvements toward muscle endurance or power performance characteristics.

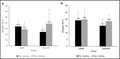

## 1 INTRODUCTION

High‐intensity interval training (HIIT) has emerged as an effective method for improving performance in elite or recreational athletes, in addition to offering health‐related fitness benefits (Gibala et al., 2012). HIIT involves speeds ranging from 80% to 100% of Maximum Aerobic Speed (MAS) (Bartlett et al., 2012) up to maximal sprinting speed (Willoughby et al., 2016), allowing for a number of repetitions to be completed with brief recovery intervals (Buchheit & Laursen, 2013) and permits an effective use of training time.

Modulation of inclination of the running surface during interval training has not been considered thus far, perhaps reflecting the fact that many sport activities normally take place on level surfaces. However, it can be appreciated that changing the running surface slope may result in distinct muscular metabolic and structural adaptations due to a variation in metabolic cost, muscle recruitment patterns, and possibly the lengths at which the involved muscles operate (Vernillo et al., 2016).

Most of the published studies introducing slope have focused on kinematic variables, for example, step length, step frequency, and contact time (Vernillo et al., 2016). Fewer studies have examined muscle strength and power functional parameters, such as peak force, time to peak force, and peak power (Ferley et al., 2014). Running on a positive slope (uphill) provides a “resistance” against the gravitational pull on the runner's body but the potential benefits on muscle power are not yet clear (Ferley et al., 2014). In well‐trained female and male distance runners performing two high‐intensity interval sessions and two continuous run sessions per week, uphill running was found to improve running economy, but not muscular power (Ferley et al., 2014). Another study employing variable slopes (up to 30% positive grade) and running speeds up to maximum for 6 weeks, revealed that uphill running can be as effective as resistance training in improving sprint start speed (Myer et al., 2007). On the other hand, downhill running has been viewed as an assistive means for improving functional power parameters (Cook et al., 2013). For example, it has been shown that combining eccentric resistance training with downhill running provided greater improvements in peak power of the countermovement jump (CMJ) and improved maximal running speed, compared to eccentric resistance alone or traditional resistance training (Cook et al., 2013).

Lower limb muscles routinely perform eccentric work as part of the stretch‐shortening cycle (Eston et al., 1995), and there is extensive literature on “isolated” eccentric muscle work, subsequent muscle damage and adaptation mechanisms (Byrne et al., 2001; Paschalis, Koutedakis, Baltzopoulos, Mougios, Jamurtas, Giakas, 2005; Paschalis, Koutedakis, Baltzopoulos, Mougios, Jamurtas, Theoharis, 2005; Paschalis et al., 2011). It is now recognized that the eccentric phase of muscle actions could benefit strength and power by improving or maintaining skeletal muscle mass, for both performance (Vogt & Hoppeler, 2014) and health purposes (Hoppeler, 2016). Past research has indicated that a slope of −10% is a metabolically optimum gradient for downhill running (Minetti et al., 1994). However, downhill high‐speed running has attracted little scientific interest to date (Gist et al. 2014), with no studies so far on high‐speed interval training (HSIT) applications. Furthermore, there is no study that has examined the effect of changes in inclination during interval running, on components of strength, such as the rate of force development and strength endurance which both are very important for success not only in sprinting but almost in any sporting activity. Therefore, the purpose of this study was to examine what the effects of high‐speed interval running would be on functional aspects of muscle performance, when using either positive or negative grade of inclination (±10%), at 90% of MAS. We hypothesized that the high metabolic load of the uphill training could promote muscle endurance performance outcomes, whereas the eccentric component of the downhill training could promote muscle strength and power performance outcomes.

## 2 MATERIALS AND METHODS

### 2.1 Participants and study design

Following a recruitment call on the university campus and in local electronic media, and a subsequent vetting interview, 14 healthy physically active adults (11M/3F) aged (23–43 years), were selected to participate in the study, after applying exclusion criteria (chronic disease, injury, medication, dieting during the last 3 months). All participants had recreational training backgrounds (e.g., running, cycling) without formally structured training regimens and no prior involvement in HIIT. After risks and benefits associated with participation in the study had been explained, participants gave their written informed consent to participate in this study, which had been approved by the University of Thessaly Ethics committee (protocol number 942/10‐12‐2014).

**FIGURE 1:**
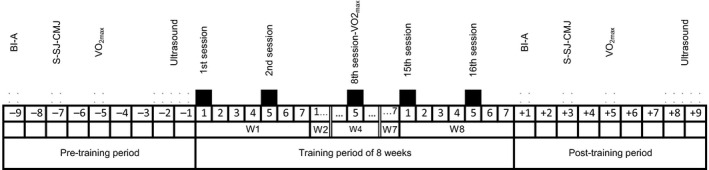
The study design. The study was divided into three periods: (a) Pre‐training, when baseline data were collected on the variables of interest (denoted by patterned squares), over a period of 8–10 days (here from −9 to −1); (b) Training, conducted over 8 weeks, during which two training sessions were performed per week (in total 16 sessions, denoted by black squares); a mid‐training re‐assessment of aerobic capacity is noted on week 4 (replacing the 8th training session, see main text); (c) Post‐training, during which post‐training data were collected, over a period of 8–10 days, in the same order as before (denoted by patterned squares). Key: Bioimpedance and other antropometry (BI‐A), Strength and jump evaluations (S‐SJ‐CMJ), Maximal aerobic capacity evaluation (VO2_max_), Muscle ultrasonography (Ultrasound), Training session (session), Weeks (W), Days are indicated by numbers, with the minus sign (−) indicating pre‐training data collection days, and the plus sign (+) indicating post‐training data collection days. The double line in the W4 box indicates that the time scale is not fully depicted for all 8 weeks

The study was divided into three periods (Figure 1): (a) Pre‐training, when baseline data were collected on the variables of interest, over a period of 8–10 days, (b) Training, during which two training sessions per week (in total 16 sessions) were conducted over 8 weeks, and (c) Post‐training, during which post‐training data were collected, over a period of 8–10 days, in the same order as before.

Participants were matched for BMI and maximal oxygen uptake and were randomly divided by a draw into either the Uphill (UG) or Downhill group (DG). They were instructed to maintain their usual nutritional and lifestyle habits and avoid any involvement in unaccustomed strenuous activities during the study. During the last week of the *pre‐training period*, they kept a diet record in order to ‐replicate it during the training period. All pre‐training tests were conducted at a convenient for each subject time of the day and were repeated at approximately the same time during the *post‐training period*. In general, volunteers were instructed to arrive for testing in a rested and hydrated state and to have avoided strenuous exercise 48 hr before testing.

During the *training period*, volunteers trained twice per week completing a total of 16 sessions over 8 weeks. In each session, they performed 10 × 30 s runs interspersed with 60 s rest intervals, at 90% of MAS at either + 10% grade (Uphill group) or −10% grade (Downhill group). The 90% of MAS was chosen, as such a speed has been found to be appropriate for HIIT (Buchheit & Laursen, 2013) (see below for MAS determination). Those gradients and the 1:2 work to rest ratio were found in preliminary work by us to be well tolerated allowing also the completion of the uphill workout (Theofilidis et al., 2014). Regarding the frequency of training and overall duration, level HIIT training with two sessions per week and a duration of as little as 6 weeks, have been shown to improve specific physiological outcomes (Barnes et al., 2013; Ferley et al., 2014; Gist et al., 2014). During the first and the last workout of the first and the last training session, expired gases were measured breath‐by‐breath to assess metabolic load (Morton & Billat, 2000). At week 4, the 8th training session was replaced by a re‐evaluation of MAS, for training running speed to be re‐adjusted accordingly, if needed.

### 2.2 Body composition

Height and weight were recorded and body mass index (BMI) in kg/m^2^ was calculated. Body composition was assessed using a whole‐body multifrequency bioimpedance spectroscopy system (BCM, Fresenius Medical Care, Germany), to estimate fat mass (FM), and Lean Tissue Mass (LTM) in kg and as % of body weight.

### 2.3 Maximum aerobic speed (MAS) determination

To identify the maximum aerobic speed (MAS) of participants, a maximal oxygen uptake (VO_2_max) test was conducted using a progressive exercise protocol on a treadmill (Stex 8025T, South Korea) as previously described (Billat et al., 1998). Briefly, after setting an initial speed, subjects completed four 3‐min stages with the speed increasing by 2 km/h (at level). Thereafter, speed was increased by 1km/h every 2 min until exhaustion. Expired gases were measured breath‐by‐breath with a gas analyzer (CareFusion Vmax encore 29, CA, USA) and averaged every 20 s. The MAS was noted at attainment of VO_2_max (Billat et al., 2000), and the 90%MAS (Buchheit & Laursen, 2013) for each volunteer was calculated and subsequently used for training. This assessment was also repeated on week 4 in case a re‐adjustment would be needed.

### 2.4 Jumping performance

On a separate day, volunteers performed three squat (SJ) and three counter movement (CMJ) jumps on a Bosco Test Jump Mat (Ergojump, MAGICA, Italy). SJs were performed from a squat position (knee angle at approximately 90°). CMJ were accomplished with a downward movement, until the subjects’ knees flexed to a visually monitored knee angle of approximately 90° (Cormie et al., 2009). Hands were fixed on the hips during all jumps (Tsiokanos et al., 2002).

### 2.5 Isometric and isokinetic dynamometry

Using a Cybex dynamometer (Norm Lumex, Ronkonkoma, NY), volunteers were placed in a seated position (90° hip angle). The torso and thigh were stabilized by straps and the lateral femoral condyle was aligned with the axis of rotation of the dynamometer's arm. For the maximum isometric knee extension and flexion trials, knee angle was set at 65° and 30°, respectively (0° = full knee extension). At each angle, volunteers executed two 5‐s all‐out efforts. The maximum voluntary contraction (MVC, in N.m) was determined from the best effort. To assess rate of force development (RFD), absolute torque values (N.m) were recorded at five time points relative to the onset of contraction for the MVC attempt (50, 100, 150, 200, and 300 ms). Impulse (N.m*s) for the 0–300 ms time fraction was calculated according to Aagaard and colleagues (2002). The onset of muscle contraction was defined as the time point at which the moment curve exceeded baseline moment by >0.4 N*m (Tillin et al., 2010).

For the isokinetic measurements, the range of motion (ROM) of the knee was set from 0–90° (0° = full knee extension). Volunteers were encouraged to perform five maximum efforts of knee extension—flexion starting from 90° knee angle at an angular velocity of 60°/sec (Kellis & Baltzopoulos, 1996). Maximum torque values and the angles at which they occurred were recorded.

Following a passive rest of 3 min, a ***fatigue protocol*** was conducted for the leg with the highest knee extension moment and only for the knee extension phase of movement. The fatigue protocol consisted of 50 maximum isokinetic efforts at 60°/sec. The effort was terminated either at completion or when volunteers could not maintain a knee moment above 50% of their maximum torque (Burdett & Vanswearingen, 1987). The total work over the sum of repetitions was also recorded.

### 2.6 Muscle architecture

Sonographs of the right vastus lateralis (VL) were taken in the middle of the muscle, at 50% of the distance from the greater trochanter to the lateral condyle of the femur (Stasinaki et al., 2015). B‐mode ultrasound images were obtained with a linear 6 mm probe (7.5 MHz, Chison Digital Color Doppler Ultrasound System, Model Q8, China 214142). Volunteers laid supine having their knees fully extended and their feet hanging off the edge in a relaxed manner. Up to three images were collected for subsequent blind analyses. An image was considered appropriate for analysis when several fascicles could be easily outlined without interruption across the image. Parameters recorded were as follows: (a) *Muscle thickness* (t), defined as the averaged distance between the superficial and deep aponeurosis measured at the two ends and at the center of each image; (b) *fascicle angle* (A), defined as the angle of insertion of muscle fascicles onto the deep aponeurosis; and (c) *fascicle length* (fL), defined as the distance between the insertions of the fascicle onto the upper and deeper aponeurosis (Stasinaki et al., 2015). In the case of fascicle lengths extending out of the field of view (as in two pre‐training cases) fL was calculated as fL = *t* × sin A^−1^ (Maganaris & Baltzopoulos, 1999). The reliability of all aforementioned measurements was significant (*p* < .026 to *p* = .000).

### 2.7 Statistical analyses

The data are presented as Mean ± standard deviation (*SD*). A 2 × 2 (group by time) mixed‐method analysis of variance, was used to assess all available data. For pairwise comparisons, effect size (ES) was determined by Cohen's *d* (small: ≥0.2, medium: ≥0.5 and large: ≥0.80). Relative or absolute changes (pre values–post values) were compared by unpaired *t*‐test. Data were analyzed with IBM SPSS Statistics v 21 for Windows (IBM Corp., Armonk, NY, USA). The level of significance was set at *p* < .05.

Power analysis was performed using the open‐source software G*Power (3.1.9.2) and was used to calculate the minimum number of participants required to achieve reasonable power (>80%). A post hoc analysis revealed that for our primary outcome RFD (whether absolute or relative), we had enough power to detect the statistical significant differences between the pre and postintervention comparisons (300ms absolute and relative to MVC torque knee extension in downhill, within groups downhill: Effect size dz = 1.3305619, total sample size = 7, *t* = 2.4469119, Power (1‐β err prob) = 0.8328973).

## 3 RESULTS

All participants completed the 8 weeks of training without missing any training session or any sprint within it and without reporting an adverse incident or injury. However, some missed data occurred for technical reasons. In those cases, the actual *N* value of the data presented is specifically reported.

The two groups demonstrated different metabolic load values pre and post‐training (Table 1). The mid‐training evaluation of MAS resulted in the re‐adjustment (increase) of running speed for five subjects from the UG and for six subjects from the DG. Body weight and BMI did not change significantly, with no statistical differences observed between the UG and DG either pre or postintervention (Table 1).

**TABLE 1:** Anthropometric and physiological characteristics of participants, pre and post‐training are presented as Mean ± *SD*

Variable	Uphill					Downhill				
	Pre‐training	Post‐training	*p*	Δ%	*d*	Pre‐training	Post‐training	*p*	Δ%	*d*
Height (m)	1.74 ± 0.1					1.77 ± 0.2				
Weight (kg)	81.3 ± 12.7	80.5 ± 10.2	.608	−0.7 ± 4.1	0.07	82.9 ± 23.8	80.9 ± 20.8	.220	−1.9 ± 3.0	0.08
BMI (kg/m^2^)	26.6 ± 3.3	26.2 ± 2.4	.418	−1.1 ± 4.1	0.12	25.1 ± 3.6	24.5 ± 2.9	.228	−2.1 ± 3.4	0.17
LTM (kg)	57.4 ± 13.8	57.9 ± 12.7	.559	1.7 ± 5.8	0.03	57.2 ± 14.6	58.6 ± 14.8	.115	2.5 ± 2.4	0.08
LTM (% BW)	70.5 ± 15.3	71.5 ± 12.4	.428	2.5 ± 6.5	0.07	69.6 ± 11.3	72.6* ± 10.9	.037	4.5 ± 4.0	0.25
VO_2_ during training (ml/kg/min)	28.4 ± 5.4	27.9 ± 4.2	.000	−1.8 ± 4.8	0.08	14.8 ± 1.5^†^	13.9 ± 1.9^†^	.000	−7.9 ± 1.7	0.51

Note

Also delta change as a percentage of baseline value (Δ%) and Cohen's *d* (*d*) are shown.

BMI, body mass index; BW, body weight; FM, fat mass; LTM, lean tissue mass.

* Denotes significantly different, *p* < .05, from pre‐training.

^†^ *p* < .05 between groups.

There were no significant differences between groups in SJ and in CMJ, pre or post‐training. Jumping performance significantly improved only for the DG (*n* = 7), in the SJ (from pre‐training values of 27.0 ± 9.2 cm to post‐training values of 29.5 ± 7.7 cm, *p* = .042, Δ% = 9.5 ± 11.7, Cohen's *d* = −0.31). Moreover, for the DG there was a tendency to improve in the CMJ (pre‐training 31.0 ± 8.2 cm to post‐training 33.1 ± 8.8 cm, Δ% = 6.2 ± 10.2, *p* = .08, Cohen's *d* = −0.22). No statistically significant changes in jumping performance were observed in the UG group (*n* = 6) in neither SJ (pre‐training 30.0 ± 9.2 cm to post‐training 30.9 ± 7.4 cm, *p* = .422, Δ% = 5.9 ± 12.8, Cohen's *d* = −0.11) nor the CMJ (pre‐training 34.7 ± 9.5 to post‐training 35.4 ± 8.2 cm, *p* = .570, Δ% = 3.3 ± 8, Cohen's *d* = −0.07).

**FIGURE 2:**
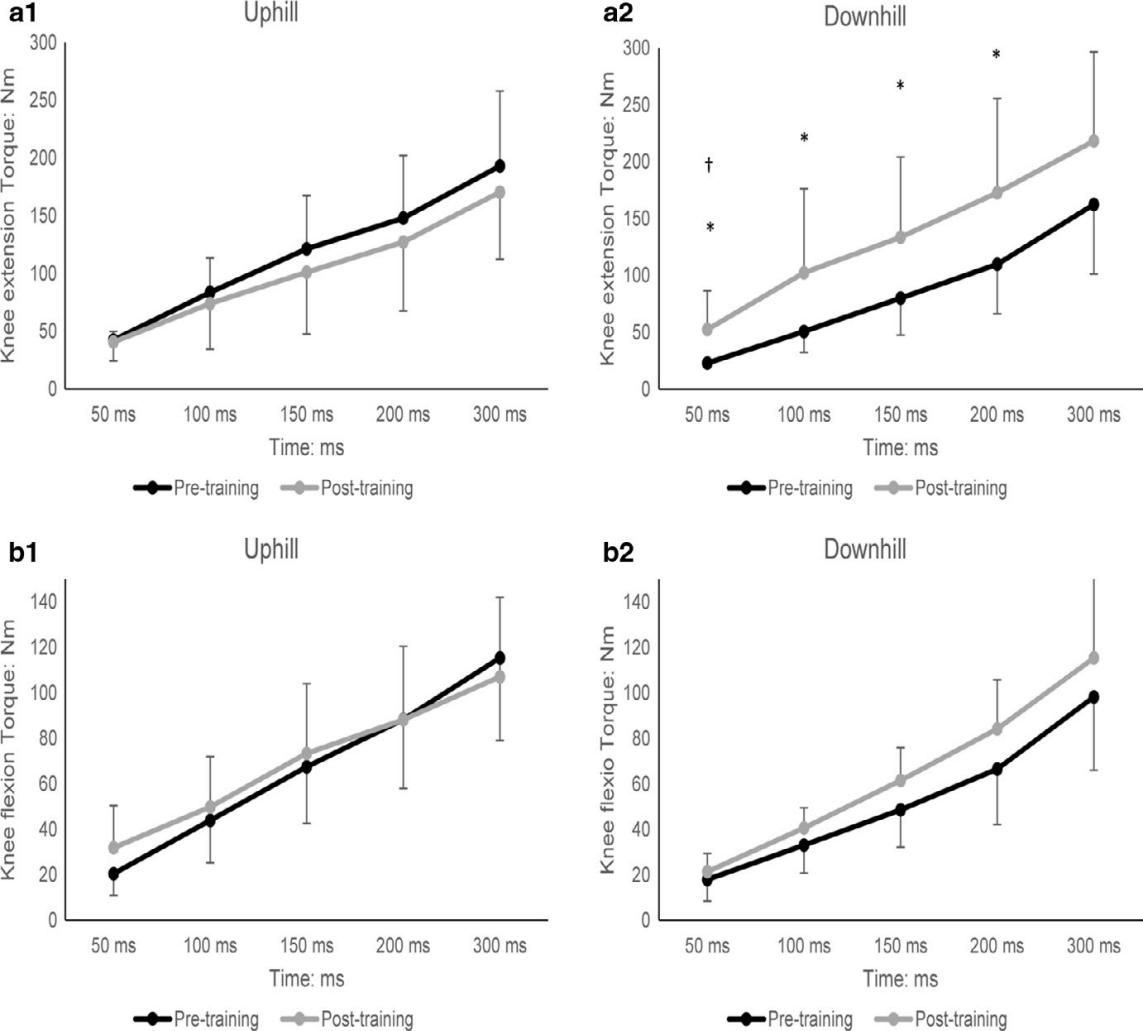
Absolute torque values at specific time points, relative to the onset of contraction for maximal voluntary contraction (MVC), are presented as Mean ± *SD*; Knee Extensors (a1) for the Uphill and (a2) for the Downhill groups, respectively; Knee Flexors (b1) for the Uphill and (b2) for the Downhill groups, respectively. *p < .05 from pre‐training, ^†^ *p* < .05 different pre‐training values between groups

**FIGURE 3:**
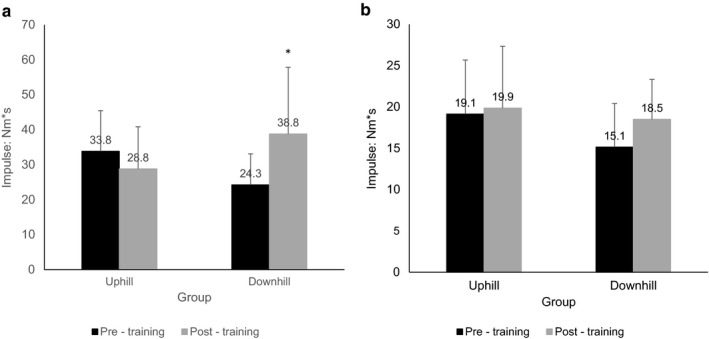
Impulse data (in N.m*s) calculated for the 0–300 ms time fraction relevant to the onset of contraction for maximal voluntary contraction (MVC), are presented as Mean ± *SD*; (a) Knee Extension; (b) Knee Flexion; Pre‐training in black and post‐training values in gray. The actual mean value number is shown.* denotes significantly different from pre‐training value. *p* < .05

The training interventions did not significantly alter the MVC of knee extension nor of flexion, in either group (Table 2). However, training improved the RFD. Specifically, the DG significantly improved the absolute knee extension torque (N.m) at the time fractions closer to the onset of contraction (i.e., at 50 and at100 ms) and tended to improve (*p* = .052 to 0.066) the remaining time fractions (see Figure 2). More specifically, for 50 ms from average toque improved from 22.9 ± 3.3 N.m pre‐training to 52.4 ± 34.1 N.m post‐training (*p* = .016); for 100 ms from 50.6 ± 18.4 N.m pre‐training to 102.2 ± 74.1 N.m post‐training (*p* = .039). Moreover, the impulse (N.m*s over 0–300 ms) improved from 24.3 ± 8.8 N.m pre‐training to 38.8 ± 19.1 N.m post‐training (*p* = .042) (Figure 3). For knee flexion, there were no statistically significant differences between pre and post‐training values of those indices in neither of the groups, only a trend for improvement for the DG (Figures 2 and 3).

**TABLE 2:** Isometric MVC and Isokinetic Peak Torque, absolute and relative to BW, values of knee extensors and knee flexors for the Uphill (UG) and Downhill (DG) groups, pre‐raining (PRE) and post‐training (POST), are presented as Mean ± *SD*

Group	Mode	Knee Extensors										Knee Flexors									
		Peak torque (Nm)					Peak torque (Nm/kg BW)					Peak torque (Nm)					Peak torque (Nm/kg BW)				
		Pre‐training	Post‐training	*p*	Δ%	*d*	Pre‐training	Post‐training	*p*	Δ%	*d*	Pre‐training	Post‐training	*p*	Δ%	*d*	Pre‐training	Post‐training	*p*	Δ%	*d*
UG (*n* = 7)	Isometric	275.3 ± 47.0	267.7 ± 46.1	0.423	−2.7 ± 4.5	0.15	4.3 ± 0.7	4.2 ± 0.6	0.846	0.1 ± 8.0	0.04	172.3 ± 39.1	156.7 ± 34.3	0.062	−8.6 ± 9.6	0.39	2.1 ± 0.3	2.0 ± 0.3	0.043	−7.4 ± 8.8	0.49
DG (*n* = 5)		301.4 ± 93.4	294.4 ± 98.7	0.529	−2.6 ± 10.6	0.07	4.7 ± 1.2	4.7 ± 1.0	0.819	3.3 ± 10.8	−0.03	173.2 ± 61.3	175.6 ± 55.1	0.794	2.7 ± 11.0	−0.04	2.1 ± 0.3	2.2 ± 0.4	0.382	6.1 ± 10.1	−0.22
UG (*n* = 7)	Isokinetic	222.3 ± 44.5	222.7 ± 46.1	0.954	0.1 ± 6.6	−0.01	2.7 ± 0.4	2.8 ± 0.4	0.584	1.5 ± 4.6	−0.09	135.3 ± 33.9	130.1 ± 34.5	0.442	−3.5 ± 13.4	0.14	1.7 ± 0.3	1.6 ± 0.3	0.692	−2.3 ± 11.6	0.11
DG (*n* = 6)		210.3 ± 73.9	212.8 ± 77.9	0.804	1.5 ± 9.4	−0.01	2.7 ± 0.5	2.8 ± 0.5	0.351	4.0 ± 8.8	−0.18	132.0 ± 50.1	135.3 ± 50.1	0.642	3.5 ± 8.0	−0.06	1.7 ± 0.4	1.8 ± 0.3	0.327	6.3 ± 9.1	−0.26

Note

Also delta change as a percentage of baseline value (Δ%) and Cohen's *d* (*d*) are shown.

* *p* < .05 from pre‐training values.

Training did not affect isokinetic knee extensors or flexors peak torque measured in absolute (Nm) or relative (%BW) terms, in any of the groups (Table 2). However, for knee flexors, there was a significant time effect on Peak Torque Angle, (*p* = .01), with post hoc tests showing that Peak Torque Angle for the knee flexors decreased significantly in both groups; there were, however, no between groups differences, pre or post‐training. More specifically, for the UG group (*n* = 7) pre‐training flexors peak torque angle changed from 28.3 ± 3.5° to 22.7 ± 4.3° post‐training, *p* = .021, Δ% = −18.9 ± 17.0, Cohen's *d* = 1.32, and for the DG (*n* = 6), it changed from pre‐training 32.0 ± 4.4° to post‐training 24.8 ± 4.0°, *p* = .006, Δ% = −22.2 ± 17.2, Cohen's *d* = 1.65).

Regarding the fatigue protocol (Table 3), the number of repetitions increased in both groups post‐training; however, only in the UG (*n* = 7), this increase was statistically significant (*p* = .047) with a ~21% improvement noted. Total work (J) done was significantly increased by ~14% for the UG (*p* = .039), whereas the DG maintained their performance in this parameter unchanged. No significant differences between the two groups were noted at pre‐training or post‐training group comparisons for the number of repetitions or total work.

**TABLE 3:** Total number of repetitions and total work for knee extension during the fatigue protocol in both training groups, are presented as Mean ± *SD*

Group	Knee Extension Fatigue Protocol									
	Repetitions					Total Work (J)				
	Pre‐training	Post‐training	*p*	Δ%	*d*	Pre‐training	Post‐training	*p*	Δ%	*d*
UG (*n* = 7)	32.6 ± 11.6	39.1 ± 14.3*	.047	21.2 ± 32.6	−0.47	5,458.6 ± 1926.4	6,300.4 ± 2,404.7*	.039	13.8 ± 21.2	−0.36
DG (*n* = 6)	31.7 ± 11.5	35.3 ± 12.4	.273	13.3 ± 20.1	−0.28	4,606.2 ± 1,482.9	5,120.0 ± 1651.3	.213	12.7 ± 14.6	−0.30

Note

Also delta change as a percentage of baseline value (Δ%) and Cohen's *d* (*d*) are shown.

* *p* < .05 from pre‐training values.

Regarding muscle architecture, no significant differences between the two groups were noted at pre‐training or post‐training group comparisons for muscle pennation angle, muscle fascicle length (fL), and muscle thickness of VL muscle pre and post‐training (Table 4). However, fL and thickness (in cm) were found to have decreased post‐training, in the downhill group (*p* = .037 and *p* = .026 respectively) (Table 4).

**TABLE 4:** Pennation angle, fascicle length (fL), and muscle thickness values in both training groups, pre and post‐training, are presented as Mean ± *SD*

Group	Pennation Angle (^o^)						Length (cm)						Thickness (cm)				
	Pre‐training	Post‐training	*p*	Δ%	*d*	Pre‐training		Post‐training	*p*	Δ%	*d*	Pre‐training		Post‐training	*p*	Δ%	*D*
UG (*n* = 7)	19.4 ± 3.8	19.8 ± 3.7	.727	2.5 ± 9.1	−0.09	8.0 ± 0.5		7.3 ± 1.3	.185	−8.5 ± 13.3	0.64	2.7 ± 0.4		2.5 ± 0.3*	.013	−6.6 ± 5.8	0.46
DG (*n* = 7)	18.4 ± 3.1	18.8 ± 3.2	.708	3.7 ± 20.5	−0.12	8.2 ± 1.7		7.3 ± 1.4*	.037	−8.9 ± 16.3	0.52	2.5 ± 0.4		2.4 ± 0.4*	.026	−6.6 ± 6.4	0.37

Note

Also, delta change as percentage of baseline value (Δ%) and Cohen's *d* (*d*) are shown.

* *p* < .05 from pre‐training values.

## 4 DISCUSSION

The purpose of this study was to examine the effects of positive or negative incline (+10% or −10% grade) applied during interval high speed running, at 90% of MAS, on selected muscle performance parameters. Our main findings were that Downhill high‐speed interval running resulted in an augmented rate of force development (RFD), as well as an increased isometric impulse and improved jumping performance. On the other hand, the Uphill high‐speed interval running improved resistance to fatigue. The above results constitute an appreciable differentiation in performance outcomes, achieved with the application of slope modulation, during interval training of a small‐time duration per session.

An important outcome in the Downhill Group, was the consistent post‐training improvement in the development of torque values of the knee extensors during the initial 300 ms of isometric exercise and the corresponding impulse. To our knowledge, this is the first study reporting such an outcome, following downhill high‐speed interval training. Our results are overall in line with the notion that power training, enhances isometric RFD (Perez‐Gomez & Calbet, 2013; Villarreal et al., 2011).

For the Downhill group, jumping ability significantly improved in the SJ (by 9.5%) and tended to improve in the CMJ (by 6.6%), something not noted in the Uphill group (see further below). To our knowledge, this is the first time that such an improvement in jumping ability is presented by just modulating the slope of the running surface during very brief intermittent exercise. Our findings are in line with a previous study reporting that the use of continuous downhill running, as an additional overspeed stimulus, brought about peak power enhancements in CMJ (Cook et al., 2013). Previously, comparable improvements in SJ and CMJ (by 9% and 13%, respectively), were reported as a result of a more demanding Stretch Shortening Cycle exercise training regime (Malisoux et al., 2006). Our findings can be linked to the enhanced RFD during isometric knee extension; an enhanced RFD has previously been associated with better jumping ability (Wilson & Murphy, 1996) and previous studies have indicated that RFD is critical for jumping performance (Chang et al., 2015).

The improvement in jumping performance for the Downhill Group is rather interesting if one considers the brevity of our training sessions, the low training frequency and the short training period. Our downhill protocol emphasized the eccentric loading of the Stretch Shortening Cycle in a moderate manner and by minimizing the risk in muscle damage, which is usually of concern when employing plyometric exercises (Macaluso et al., 2012). Furthermore, past studies demonstrated comparable improvements in jumping performance by employing longer training durations (up to 12 weeks) with higher training frequency (e.g., 4 sessions/week), higher intensity (e.g., based on high percentages (120%) of 1‐RM) (Cook et al., 2013), higher loads and/or more demanding tasks (e.g., single‐leg hurdle jumps) (Malisoux et al., 2006), or more complex designs (e.g., with a combination of weights and plyometric training) (Chatzinikolaou et al., 2018; Villarreal et al., 2011).

Regarding the Uphill Group, jumping ability did not change. These results are in accordance with other studies showing no changes in jumping performance after level surface HIIT (Mueller et al., 2015). The lack of change in jumping ability was also mirrored in the unchanged RFD values for the Uphill group; such results are also in accordance with previously published reports indicating a reduction in RFD in knee extensors in endurance‐trained subjects, with the use of HIT (Mueller et al., 2015) or classical endurance training methods (Farup et al., 2014).

Notably, the Uphill Group improved their ability to resist fatigue by ~20% and produced more work by 15%. Such an outcome suggests that interval uphill running might serve as a strength‐endurance training method. It is worth mentioning that the Downhill Group also improved their total number of repetitions and the total work produced during the fatigue protocol, but to a lesser extent, suggesting that interval downhill running may provide a sufficient stimulus for maintaining if not improving strength endurance characteristics. Therefore, it is intriguing to claim that the overall improvement in the ability of both groups to resist fatigue derived from peripheral muscular adaptations, toward more fatigue resistant fiber properties (Bogdanis, 2012).

As this is the first study comparing uphill versus downhill high‐speed interval running, we cannot make direct comparisons with the existing literature regarding strength gains. In a study with a different design, using continuous downhill (–10% slope) running ranging from 5 up to 20 min per session, knee extensors peak torque increased (Toyomura et al., 2018). This suggests that the total duration of our training stimulus, of only 5‐min actual downhill exercise per session, might have been inadequate to allow changes in isokinetic peak torque. Additionally, strength gains are highly specific to the mode of contraction and velocity of movement, and perhaps their detection may obscured if those factors cannot be considered during testing (Roig et al., 2009). Regarding the Uphill Group, our results are in line with those studies demonstrating no changes in maximal concentric leg extension power after HIIT conducted at level surface (Jakobsen et al., 2012) or uphill (Ferley et al., 2014). Overall, the results of this study showed that peak isokinetic and isometric torque did not improve with brief uphill or downhill interval training.

In this study, the knee flexors’ peak torque angle curve shifted toward longer muscle lengths for both groups. It has been proposed that eccentric training results in sarcomerogenesis (Douglas et al., 2017) and shifts the peak of the torque–angle curve toward longer muscle lengths, which can prove important for injury prevention and improvement of athletic performance (Brughelli & Cronin, 2007). In our study, the peak–torque angle curve shift may be attributed to the different pattern of knee flexors’ activation during running on sloping surfaces. For example, changes in lower limb electromyographic activity have been reported as a result of graded uphill running (Vernillo et al., 2016). Furthermore, it has been shown that in eccentric training, the starting muscle‐tendon unit length and muscle activation time are the primary stimuli for sarcomerogenesis and not muscle strain per se (Butterfield & Herzog, 2006). We are not in a position to verify if such adaptations were at play in this study, and thus would explain the peak–torque angle curve shift, however, further work is warranted in this direction.

We reported a small but significant reduction in fascicle lengths with training, without changes in pennation angle, and a reduction in muscle thickness. Due to the design of our study, we cannot make direct comparisons with the literature, however, the pennation angle and fascicle lengths recorded by us were within the range of previously reported values for vastus lateralis (Friederich & Brand, 1990). Previously a nonsignificant reduction in fascicle lengths has been reported after continuous level endurance running (Murach et al., 2015). In theory, shorter muscle fascicle lengths, as a training adaptation, could benefit muscle endurance through improved mechanical efficiency (Murach et al., 2015). Interestingly, endurance‐trained runners have been shown to have shorter VL fascicle lengths and muscle thickness compared to sprinters (Abe et al., 2000). In that direction, the reduction in muscle thickness observed in both groups could be seen as a training adaptation toward muscle endurance, as reduced muscle cross‐sectional area with endurance training results in improved efficiency (Harber et al., 2004). Still, as the margin of error in such measurements can be large (e.g., ~7% for fascicle lengths, (Muramatsu et al., 2002) and sonographs disregard the curvature of hypertrophied muscle (Blazevich, 2006), one should interpret these findings with caution, and further work, is needed to characterize how interval training at either positive or negative incline could affect muscle architecture.

While this study constitutes a first in the literature, pointing to novel applications of HIIT and HSIT protocols by modulating the slope of running, it has methodological limitations that need to be acknowledged and addressed in the future. As already discussed the number of participants and the unequal mixed gender in our groups may have affected the power of our results; despite this, RFD and jumping performance improved with the downhill protocol, whereas fatigue tolerance increased in both groups, but more so with the one following the uphill protocol. Past literature has indicated that the mode of testing, the duration of training, and the load per se are critical factors to allow manifestation of strength gains. Here, the short duration of the training period and the training load per se (an overall duration of the training stimulus of only 5 min per session) may be considered a limitation. However, despite the brief actual exercise load per session, we were able to identify measurable performance adaptations.

The brief time/effort investment of the implemented protocols can be viewed as an advantage of this approach for the future development of efficacious training. Future research should explore in more detail the optimization of grade, duration, and load as well as explore alternative testing procedures (e.g., multijoint kinetic tasks such as squat or leg press). We envisage that slope modulation of HIIT/HSIT protocols could be used for targeted short‐term conditioning of professionals (such as military personnel), for maintaining general conditioning of otherwise competitive athletes whose schedules are often overloaded (such as footballers) and/or maintaining specific fitness characteristics of the competitive amateur athlete who (due to work and family obligations) may have limited time to train during the week.

In conclusion, this study examined, for the first time, how opposing slopes of the running surface could differentiate the effects of high‐intensity/speed interval training on functional aspects of muscle performance. After 8 weeks of training, Uphill interval running improved strength endurance characteristics, whereas Downhill interval running improved jumping ability, rate of force development, and impulse; the latter muscle power benefits were favorably comparable to such improvements achieved by traditional, far more complex and demanding, muscle strength, and power interventions. Our results thus show that slope modulations can help further specify benefits of HIIT implementation, and support the introduction of slope as a new factor whose manipulation one should consider, along running speed, and work to rest ratio, for the prescription of HIIT.

## CONFLICT OF INTEREST

The authors declare that they have no competing interests.

## AUTHORS’ CONTRIBUTIONS

CK, GB, and GT conceived the study. CK, GT, GB, and GKS designed the study. GT, GKS, ASK, AK, TT, and CK collected data. GT, AK, ASK, TT, YK, GKS, and GS analyzed data. GT, GKS, and CK created figures and tables. GT and CK co‐wrote the manuscript. CK, GB, YK, GKS, and GS edited the manuscript. All authors have read and approved the final version of the manuscript and agree with the order of presentation of the authors.
